# Can immunotherapy reinforce chemotherapy efficacy? a new perspective on colorectal cancer treatment

**DOI:** 10.3389/fimmu.2023.1237764

**Published:** 2023-09-18

**Authors:** Xing He, Huanrong Lan, Ketao Jin, Fanlong Liu

**Affiliations:** ^1^ Department of Gastroenterology, Jinhua Wenrong Hospital, Jinhua, Zhejiang, China; ^2^ Department of Surgical Oncology, Hangzhou Cancer Hospital, Hangzhou, Zhejiang, China; ^3^ Department of Colorectal Surgery, Affiliated Jinhua Hospital, Zhejiang University School of Medicine, Jinhua, Zhejiang, China; ^4^ Department of Colorectal Surgery, the First Affiliated Hospital, Zhejiang University School of Medicine, Hangzhou, Zhejiang, China

**Keywords:** colorectal cancer, tumor treatment, chemotherapy, immunotherapy, cell therapy

## Abstract

As one of the main threats to human life (the fourth most dangerous and prevalent cancer), colorectal cancer affects many people yearly, decreases patients’ quality of life, and causes irreparable financial and social damages. In addition, this type of cancer can metastasize and involve the liver in advanced stages. However, current treatments can’t completely eradicate this disease. Chemotherapy and subsequent surgery can be mentioned among the current main treatments for this disease. Chemotherapy has many side effects, and regarding the treatment of this type of tumor, chemotherapy can lead to liver damage, such as steatohepatitis, steatosis, and sinus damage. These damages can eventually lead to liver failure and loss of its functions. Therefore, it seems that other treatments can be used in addition to chemotherapy to increase its efficiency and reduce its side effects. Biological therapies and immunotherapy are one of the leading suggestions for combined treatment. Antibodies (immune checkpoint blockers) and cell therapy (DC and CAR-T cells) are among the immune system-based treatments used to treat tumors. Immunotherapy targets various aspects of the tumor that may lead to 1) the recruitment of immune cells, 2) increasing the immunogenicity of tumor cells, and 3) leading to the elimination of inhibitory mechanisms established by the tumor. Therefore, immunotherapy can be used as a complementary treatment along with chemotherapy. This review will discuss different chemotherapy and immunotherapy methods for colorectal cancer. Then we will talk about the studies that have dealt with combined treatment.

## 1 Introduction

According to GLOBOCAN data, colorectal cancer (CRC) is the third most common cancer in men and the second in women. It is also known that its mortality is higher in men than in women (1). This type of cancer has the highest incidence rate in Europe, Australia, North America, and New Zealand (2). Instead, the incidence of this disease in Africa and South-Central Asia is the lowest among others (3). It seems that diet, environment, and genetics are the most influential factors in the susceptibility to this disease. Colorectal cancer (CRC) is divided into two groups based on DNA stability, mutation, and repair (4). The first type is characterized by DNA mismatch repair (dMMR), a high level of microsatellite instability (MSI-H), and reduced expression of beta 2 microglobulin (B2MG) (presence in the MHC-1 structure and contributing to its stability at the cell surface) (5). The second type has stable microsatellites (MSI-L) and is mismatch-repair-proficient (pMMR) (6). In addition, these two types of CRC differ in immune checkpoint ligands expression level, MSI-H tumors have high expression, and MSI-L type has low expression of immune checkpoint ligands (7, 8). Due to this characteristic, the two types of CRC respond differently to different immunotherapy treatments and knowing the type of colorectal cancer is essential for immunotherapy. As expected, cancers with instability in DNA experience fewer repairs and stably undergo mutations (9, 10). These mutations lead to the production of protein antigens (presented and new peptides on the surface of tumor cells) called neoantigens which have better immunogenicity than tumors with a lower mutation and show a high level of DNA repair (11).

Also, in another type of classification, four consensus molecular subtypes (CMSs) with a distinguishing feature are described: CMS1 (microsatellite instability immune, 14%), hypermutated, microsatellite unstable and robust immune activation; CMS2 (canonical, 37%), epithelial, marked WNT and MYC signaling activation; CMS3 (metabolic, 13%), epithelial and evident metabolic dysregulation; and CMS4 (mesenchymal, 23%), prominent TGF-β activation, stromal invasion, and angiogenesis. Samples with mixed features (13%) possibly represent a transition phenotype or intratumoral heterogeneity. So knowing the type of CRC is very important in choosing the proper treatment (12).

In addition, the results of studies have shown that chemotherapy as monotherapy cannot completely remove the tumor (13). Sometimes, even after surgery, the cancer recurs and can disrupt the patient’s life (14). Therefore, the use of simultaneous and combined treatments is suggested. One of the most important treatments that have received much attention today is tumor immunotherapy, which has shown promising results (15). Immunotherapy includes various treatments based on antibodies and T cell transfer; these are among the most critical cells used in CRC immunotherapy, and they are used in three ways, expanded without any change, TCR genetic manipulation, and CAR-T cell application (16). Also, dendritic cells (DCs) can be used to treat various tumors (17). Antibodies (Ab) used in the treatment of tumors target multiple pathways of tumor progression, including angiogenesis, tumor growth, metastasis, and immune suppression mechanisms, which have been very promising (18, 19). One group of the most important antibodies used in treating CRC (even in MSS type) is immune checkpoint blockers, which suppress the pathways developed by tumor cells to suppress the immune system (20, 21). In this article, we will first talk about approved chemotherapy and their combined uses. Then we will discuss the various available immunotherapies. Finally, we will discuss the talk about studies that use the combination of chemotherapy and immunotherapy for CRC treatment.

## 2 Colorectal cancer chemotherapy

Chemotherapy is one of the first treatment strategies after tumor diagnosis (22). However, this type of treatment should be personalized according to the patient’s tumor characteristics (23). Among the elements that should be checked include the general state of health, biology of the tumor (its aggressiveness), side effects of the chemotherapy regimen, left and right laterality and the primary location of the tumor, the drugs currently taken, other co-morbidities, and mutation status of important genes in colorectal cancer, including genes related to RAS and BRAF in tumor cells (24, 25). Mutations of RAS and BRAF genes can activate cell signaling pathways related to cell proliferation and differentiation (26). Investigating these types of mutations is very important because they can lead to resistance of cancer cells to treatment with EGFR inhibitors (27). Some chemotherapy drugs that the has approved include 5-fluorouracil, irinotecan, oxaliplatin, trifluoridine-tipiracil, and capecitabine (28, 29). These drugs exert their anti-tumor effects by different cells affecting growth pathways. However, using these drugs is not without harm to healthy cells, and they have different side effects, which we will discuss later.

### 2.1 Colorectal cancer treatment by 5-fluorouracil (5-FU)

5-FU (uracil analog) is an anti-metabolite drug (30) that replaces fluorine with hydrogen at the C5 position of uracil and ultimately leads to the formation of adenine-uracil/5-FU base pairs (31). 5-FU, after the entrance to a cell by the facilitated transport mechanism, converted intracellularly to several active metabolites, including 1) fluorouridine triphosphate (FUTP), 2) fluorodeoxyuridine triphosphate (FdUTP), and 3) fluorodeoxyuridine monophosphate (FdUMP) (31, 32). 5-FU normally exerts its antitumor effects (mediated by active metabolites) by three mechanisms. This drug can inhibit thymidylate synthase (TS) (33). This action disrupts intracellular deoxynucleotide pools required for DNA replication and cell proliferation (34). In addition, this drug can replace more than 40% of cellular RNA uracils, which can lead to the disruption of RNA synthesis (35). Also, this drug can be attached to cellular DNA after its anabolism inside the cell, leading to DNA fragmentation (36).

5-FU has been used orally or intravenously since 1990 (37, 38). However, due to the considerable variation in pharmacokinetics and unpredictable absorption, its oral use is not recommended and has been abandoned (39). The results of studies show that only 3% of 5-FU (prescribed dose) becomes toxic to cancer cells through anabolic actions (40). Although most of the administrated amounts of 5-FU are catabolized in the liver through the activity of the dihydropyridine dehydrogenase enzyme and turn into a non-toxic and inactive metabolite (41). Also, studies have shown that 20% of 5-FU prescribed through infusion is excreted through urine directly and without any change (42).

One of the drugs that can be used alongside 5-FU and increase its therapeutic efficiency is leucovorin (LV) (43, 44). The simultaneous use of these two drugs leads to an increase in the survival of patients, a decrease in side effects (chemoprotection), and an increase in the therapeutic potential of 5-FU (45). Side effects associated with the therapeutic use of 5-FU are divided into three categories. The first category is related to the effects of this drug on general conditions, including fatigue, mucositis, vomiting, diarrhea, nausea, fever, and stomatitis (46). The second category is its effects on immune system cells, blood cells, and other healthy cells, and it includes neutropenia, anemia, leukopenia, thrombocytopenia, skin rashes, and neuropathy (47). The third category comprises neurological abnormalities, including changes in cognitive function and cerebellar ataxia, which occur less often than the previous two categories (48). Cardiotoxicity (although its pathogenesis has not been completely determined) is one of the side effects of chemotherapy with 5-FU, which rarely happens, but can seriously affect the patient’s health (49).

Also, dihydropyrimidine dehydrogenase (DPYD), as a highly polymorphic gene, may be affected the treatment outcome in fluoropyrimidine-based treatments (50, 51). The product of this gene is the rate-limiting enzyme in fluoropyrimidine metabolism, or dihydropyrimidine dehydrogenase (DPD), whose function defects can lead to the accumulation of toxic metabolites from fluoropyrimidine (51). Investigations show that in patients with DPYD pathogenic variants receiving the standard dose of fluoropyrimidine chemotherapy, the risk of death due to treatment increases (52). Therefore, considering that the variants of this gene have been identified to a certain extent, it is recommended to investigate the variants of this gene before approving this type of drug. The results have shown that c.2194G>A is the most common polymorphism associated with DPYD, which is also associated with neutropenia (53). However, there is still no suitable single-nucleotide polymorphism (SNPs) panel to investigate DPYD variants, requiring more investigations.

The results of various studies show that this drug has multiple effects, including oxidative stress that causes myocardial damage, coronary artery spasm, ischemia caused by impaired oxygen transfer from red blood cells, and endothelial damage leading to thrombosis, and it causes cardiotoxicity and heart tissue damage (54). However, considering the therapeutic effects of 5-FU regardless of its side effects, this drug is being used in combination with other drugs in many clinical trials (**Table 1**).

**Table 1:** Example of chemotherapy drugs application in combination with Fluorouracil in clinical trials.

Study name	Intervention Model	Estimated Enrollment	Drugs	Phase	Date	NTC number	Major findings
5FU/LV, Irinotecan, Temozolomide, and Bevacizumab for MGMT Silenced, Microsatellite Stable Metastatic Colorectal Cancer.	Sequential Assignment	18	1. Bevacizumab 2. Irinotecan 3. Leucovorin 4. 5-Fluorouracil 5. Temozolomide	Phase 1	2020	NCT04689347	Recruiting
Metformin and 5-fluorouracil for Refractory Colorectal Cancer	Single Group Assignment	50	Metformin and Fluorouracil	Phase 2	2013	NCT01941953	1. Metformin has anti-tumor activity (55). 2. Metformin decreases 5-FU side effects.
mFOLFOX6 Combined With Dalpiciclib in Patients With Metastatic Colorectal Cancer (FIND)	Single Group Assignment	18	1. Dalpiciclib 2. Oxaliplatin injection 3. Calcium folinate 4. 5-fluorouracil	Phase 2	2022	NCT05480280	Recruiting
Study of Magrolimab Given Together With FOLFIRI/BEV in Participants With Previously Treated Advanced Inoperable Metastatic Colorectal Cancer (mCRC)	Parallel Assignment	135	1. Magrolimab 2. Bevacizumab 3. Irinotecan 4. Fluorouracil 5. Leucovorin	Phase 2	2022	NCT05330429	Recruiting
Metastatic Colorectal Cancer (RAS-wildtype) After Response to First-line Treatment With FOLFIR Plus Cetuximab	Parallel Assignment	550	1. Irinotecan 2. Folinic Acid 3. 5-Fluorouracil 4. Cetuximab 5. Bevacizumab 6. Capecitabine 7. regorafenib 8. Irinotecan 125mg 9. Cetuximab wkly	Phase 3	2016	NCT02934529	Recruiting
Study in mCRC Patients RAS/BRAF wt Tissue and RAS Mutated LIquid BIopsy to Compare FOLFIRI Plus CetuxiMAb or BevacizumaB	Parallel Assignment	280	1. Bevacizumab 2. Cetuximab 3. 5-Fluorouracil 4. Irinotecan 5: Calcium levofolinate	Phase 3	2021	NCT04776655	Recruiting
Systemic Chemotherapy Plus HAI(FUDR) vs Systemic Chemotherapy Alone For CRCLM	Parallel Assignment	288	1. FUDR 2. Oxaliplatin 3. Leucovorin 4. 5-Fluorouracil	Phase 3	2018	NCT03500874	Recruiting
A Randomized Trial of Avastin + Gemcitabine + 5-Fluorouracil (5FU)/Folinic Acid Versus Avastin + Oxaliplatin + 5FU/Folinic Acid in Metastatic Colorectal Cancer	Parallel Assignment	84	1. Gemcitabine 2. Avastin 3. 5-FU/folinic acid 4. Oxaliplatin	Phase 2	2009	NCT00192075	1. Folinic acid, 5-fluorouracil, gemcitabine (FFG), and FOLFOX4 were generally well tolerated. 2. FGG has no potential advantage over 5-FU/folinic acid (56).

### 2.2 Colorectal cancer treatment by capecitabine

Despite the success of using 5-FU in treating colorectal cancer, due to the short half-life of this drug (requiring multiple injections) and its rapid clearance from the body, researchers are looking for a way to redesign and use the therapeutic advantages of this drug (57). The results of researchers’ efforts in 2009 led to the discovery of capecitabine, a prodrug of 5-FU, which has advantages over 5-FU (58). Unlike 5-FU, this drug is oral, and after being absorbed through the patient’s digestive system, it is converted into 5-FU by successive enzymatic reactions, first in the liver and then in the tumor site (3 reactions) (58, 59). Thymidine phosphorylase (TP) is expressed in higher concentrations in neoplastic tissue (60, 61). This enzyme mediates capecitabine final conversion from 5’-deoxy-5-fluorouridine to 5-FU; therefore, the production of the active form of the drug is preferably done in tumor tissue (62, 63). Also, the results of studies have shown that the level of TP expression in tumor tissue is increased after exposure to radiotherapy and cytotoxic agents to help tumor eradication synergistically (64).

### 2.3 Colorectal cancer treatment by irinotecan (IRI)

IRI is a water-soluble semi-synthetic chemotherapy drug derived from camptothecin, which was approved for the treatment of lung cancer, cervical cancer, and ovarian cancer in Japan in 1994 (65, 66). This drug is also used to treat metastatic colorectal cancer (67). The results of clinical trials showed that the combination of IRI with 5-FU/LV can significantly increase the survival of patients compared to the 5-FU/LV receiving group (68). Also, this drug is used in combination with oxaliplatin and can help to improve patients’ conditions by inhibiting metastasis (69–71). Irinotecan has an acceptable tolerability profile, increases the duration of treatment, is not associated with cumulative toxicity in patients with metastatic CRC, and leads to improved patient survival and quality of life (QOL) (66). Various studies have shown that exposure to ethyl-10-hydroxy-camptothecin (SN38), the active metabolite of irinotecan, shows different results in different people and can lead to severe toxicity in patients receiving the drug (72). Also, the drug dosage should be strictly controlled in some patients, including patients with severe renal failure and patients with UDP-glucuronosyltransferase 1A1 (UGT1A1) polymorphism (73).

IRI exerts its antitumor function usually by inhibiting topoisomerase I (74). However, the results of studies show that this is not the only functional mechanism of IRI. Cells exposed to IRI experience extensive gene expression changes. SN38 interacts with the various vital proteins for the cell, including BCL-xL (75), which has an anti-apoptotic role, up-regulation of FAS (76), mouse double minute 2 homolog (MDM2) involved in TP53-mediated cell death (77), and activation of MAPK signaling pathway can lead to increased cancer cells apoptosis (78). The excretion and pharmacokinetics of IRI depend on several factors, such as dosage, liver function status, age, administration time, and gender (79).

One of the side effects of IRI application is neutropenia (more common in women than in men) in receiving patients (80). Several side effects with the use of this chemotherapy drug, including delayed and severe diarrhea, abdominal pain, steatohepatitis, metabolic changes in patients’ plasma (accumulation of acylcarnitines, nucleobases, and certain amino acids in plasma), similar cholinergic symptoms, oxidative stress in the liver and sweating they experience (81).

### 2.4 Colorectal cancer treatment by oxaliplatin (Ox)

Oxaliplatin is a platinum-derived drug used to treat metastatic colorectal cancer distributed throughout the body by binding to plasma proteins (82). About half of the Ox injected into patients is eliminated through urine, but its excretion in feces is insignificant (clearance unrelated to liver function) (83). Most of the side effects of this drug are related to the release of platinum-active species (84) and its binding to DNA sequences (usually to GA or GG), which prevents DNA repair and synthesis in healthy cells (85). Currently, Ox is not usually used alone in treating colorectal cancer, but this drug is combined with other chemotherapy and biological drugs (83). For example, in three randomized clinical trials, adding oxaliplatin to a regimen of leucovorin, capecitabine, and fluorouracil resulted in a 20% reduction in disease recurrence (86).

### 2.5 Colorectal cancer treatment by trifluridine-tipiracil

As it is clear from the name of the drug trifluridine/tipiracil, this drug consists of two parts. Trifluoridine is a thymidine-related nucleoid analog and replaces thymidine in DNA (87). This is while tipiracil strengthens the function of trifluridine by inhibiting the thymidine phosphorylase enzyme (88). Tipiracil leads to trifluridine replacement in DNA by preventing thymidine bases and ultimately prevents cell proliferation (87). The most common side effects associated with the use of this drug in metastatic colorectal cancer patients included neutropenia, anemia, thrombocytopenia, and leukopenia (89). Trifluoridine in this medicine, similar to what is seen in the therapeutic use of 5-FU, is converted into a monophosphorylated form by thymidine kinase (90). Still, unlike 5-FU, the monophosphorylated form of trifluorothymidine inhibits the activity of this enzyme by binding to the active site of the thymidylate synthase enzyme (90). It leads to cytotoxicity and non-production of thymidine by this enzyme. Subsequent phosphorylation by thymidine kinase produces trifluridine triphosphate, readily incorporated into the DNA of tumor cells (in place of thymidine bases), interferes with DNA function, and inhibits tumor growth (91). In clinical applications, this drug leads to the inhibition of tumor growth in a dose-dependent manner (92). The results of new studies show that this drug has high therapeutic efficiency, is very easy to use, and has fewer side effects than other chemotherapy drugs (93).

## 3 Colorectal cancer combinational chemotherapy

In many combination treatments related to colorectal cancer chemotherapy, 5-FU or capecitabine are usually the leading drugs. The results of the studies show that the therapeutic effects of capecitabine have more promising effects compared to the combined treatment of LV/5-FU (94, 95). However, chemotherapy regimens based on 5-FU have been designed over many years (96).

Among these treatments, we can mention FOLFOX, which includes folinic acid, 5-FU, and oxaliplatin (97, 98). The therapeutic efficacy of this combination is greater than the single use of oxaliplatin and 5-FU, but its primary mechanism is unclear (99). Today, this combination seems more effective in affecting the intestinal microbiota (100, 101). The 16S rRNA gene sequence analysis from new studies has shown that the abundance of *Akkermansia muciniphila* increases significantly in patients receiving the FOLFOX combination (102). Further studies on *Akkermansia muciniphila* showed that dipeptides containing branched-chain amino acids (BCAA) are one of the main factors in increasing the anticancer activity of the mentioned compound (102). The results of various studies have shown that CAPOX (folinic acid, capecitabine, oxaliplatin) and FOLFOX are the most effective regimens in treating advanced colon cancer (103), with the difference that in the CAPOX regimen, the infusion of 5 FU is replaced by an oral derivative of capecitabine (104).

FOLFIRI is one of the other combined chemotherapy options that consists of 400 mg/m^2^ 5-FU (day one iv bolus), 600 mg/m^2^ 5-FU (days 1 and 2 iv by ci), and 180 mg/m^2^ irinotecan (day 1 iv) and are repeated every two weeks (105, 106). Due to the low therapeutic efficiency of 5-FU (about 10 to 15%), combination treatments of chemotherapy drugs, including FOLFIRI, are used, which leads to an increase in efficiency of up to 45% (105). The results of new studies show that the treatment outcome can be predicted based on the profile of the immune system cells of a person with colon cancer, especially the Treg/TH ratio (107). So patients with a higher Treg/TH ratio respond better to FOLFIRI treatment than patients with a lower ratio (108). Also, a specific decrease in the population of regulatory T cells was observed in patients receiving FOLFIRI (109). Therefore, it seems that some patients’ high number of regulatory T cells does not have the immunological pressure to make the tumor more resistant. When these patients are faced with treatment, they respond to it in a better way (107).

The combination of capecitabine and irinotecan (CAPIRI) is another chemotherapy combination used less in studies than other combinations (110). However, the various results that have used chemotherapy compounds have acknowledged that these compounds cannot eradicate tumor cells. Additional treatments, including biological or immunological options, are needed in their cases. **Table 2** summarizes some approved combined and single drug-based chemotherapy regimens.

**Table 2:** Summary of some approved combined and single drug-based chemotherapy regimens.

Chemotherapy regimen	Injection program	Type	Components	Ref.
Lokich	Daily IV injection	Single	5-FU (300 mg/m2)	(111)
TTD	IV infusion daily for 5 days, repeated every 28 days	Combined	1. Bolus leucovorin (200 mg/m2) 2. 5-FU (370 mg/m2)	(112)
FOLFOX-4	Programed infusion in 2 day	Combined	1. Leucovorin (200 mg/m2, day 1,2) 2. 5-FU bolus (400 mg/m2, day 1,2) 3. Continuous infusion 5-FU (600 mg/m2 for 22 h, day 1,2) 4. Oxaliplatin (85 mg/m2 day 1)	(113)
FOLFOX-6	Programed infusion in 2 day	Combined	1. Leucovorin (400 mg/m2 IV, day 1) 2. 5-FU (400 mg/m2 IV bolus on day 1; then a continuous infusion of 1,200–1,500 mg/m2/day × 2 days) 3. Oxaliplatin (100 mg/m2 IV, day 1)	(113)
FOLFIRI	Programed infusion in 2 day	Combined	1. Leucovorin (400 mg/m2 IV, day 1) 2. 5-FU (400 mg/m2 IV bolus, day 1; then a continuous infusion of 1,200–1,500 mg/m2/day × 2 days) 3. Irinotecan (180 mg/m2 IV, day 1)	(114)
FOLFOXIRI	Programed infusion in 2 day	Combined	1. Irinotecan (165 mg/m2 over 60 min) 2. Oxaliplatin (85 mg/m2) 3. Leucovorin (200 mg/m2 over 120 min) 4. 5-FU (3200 mg/m2 continuous infusion for 48 h)	(115)
Capecitabine	Twice daily, days 1–14	Single	Capecitabine (1000–1250 mg/m^2^)	(116)
CAPOX/XELOX	Twice daily, days 1–14	Combined	1. Capecitabine (1,000 mg/m2 twice daily PO for 14 days) 2. Oxaliplatin (130 mg/m2 IV, day 1)	(117)
mXELIRI	Daily, days 1–14	Combined	2. Irinotecan 200 mg/m2 (Day1) 1. Capecitabine 1,600 mg/m2/day (Day 1–14),	(118)

## 4 Biological treatments for colorectal cancer

Many biological treatments are directly related to immunological treatments because the responsible for their function is an antibody or its derivatives; however, in this section, we will talk about drugs that can affect tumor biology. Currently, three biological agents, which are also considered immunological agents, include bevacizumab, an anti-VEGF-A antibody; cetuximab, an anti-EGF receptor antibody; and panitumumab, an anti-EGFR antibody, which has been approved for the first-line treatment of metastatic colorectal cancer (119). These antibodies prevent cancer cell proliferation by blocking growth receptors’ function. In CRC patients who have mutated RAS in association with BRAF V600E mutation, along with chemotherapy, bevacizumab is the only agent that can lead to increased treatment efficiency (120). In addition, panitumumab and other anti-EGFR antibody applications are limited to tumors with mutations in RAS (121). Therefore, knowing the tumor type and properties can help choose the appropriate treatment with biological agents.

In addition to approved biologic therapies, researchers are currently searching for new biologic drugs to treat CRC. One of the most critical aspects of various tumors is mesenchymal-epithelial transition (122). This phenomenon is facilitated by the binding of hepatocyte growth factor (HGF) to its tyrosine kinase receptor called c-MET (119, 123). Blocking this pathway is particularly important because of the significant role of mesenchymal-epithelial transition in metastasis (124). Therefore, the biological drugs under investigation are essential for this path. Onartuzumab, Tivantinib, Savolitinib, and Cabozantinib can be mentioned among these drugs (125). However, the use of biological treatments is not without side effects. For example, using bevacizumab may have serious side effects such as proteinuria, impaired wound healing, hypertension, arterial (but not venous) thromboembolic events, bowel perforation, bleeding, and leukoencephalopathy.

Due to the considerable overlap of biological treatments with immunological treatments, they are usually grouped together. However, because these treatments directly affect the biology of tumor cells and do not affect the immune system’s responses, they are known by this name.

## 5 Colorectal cancer immunotherapy

The results of previous studies have shown that increasing the penetration of T cells into the colorectal tumor can control tumor growth (126). After identifying the MHC-peptide complex and in the presence of co-stimulatory signals, T cells identify tumor cells and destroy them using different methods, including releasing granules (cytotoxic T lymphocytes) (127). In a constant challenge, tumor cells are established in the body and destroyed by the immune system’s cells (128). Tragedies begin when the immune cells cannot destroy malignant or neoplastic cells. An important point about the tumor is its microenvironment. As the cancer progresses, it evolves to suppress the immune system’s responses (129, 130). Different types of tumors use different mechanisms, but for example, they can reduce the expression of MHC-1 molecules (131), reduce the expression of co-stimulatory molecules (132), increase the expression of growth factors (133), and increase the expression of anti-inflammatory cytokines and inhibitory surface molecules (CTLA-4, TIM-3, PD-1, PD-L1, LAG-3, and A2AR) (134–136). In addition, tumor cells secrete extracellular vesicles (EVs) into the tumor environment, reaching the immune system cells and disrupting their functions (137). Therefore, even if tumor cells are destroyed by chemotherapy, there must be a competent immune system that cleans the dead cells and also eliminates the remaining tumor cells with its abilities.

For this reason, tumor immunotherapy’s importance has increased daily (**Table 3**). Tumor immunotherapy can be divided into several parts. Passive immunotherapy occurs through the transfer of antibodies (native or engineered) mRNA (151), and cytokines (152, 153), and active immunity, which is usually performed through transferring cells related to the immune system, including dendritic cells (DCs) (154) and T cells (155).

**Table 3:** Examples of FDA-approved novel therapeutics in colorectal cancer.

No	Drug name	Trade name	Properties	Disease	Date of approval	Ref.
Chemotherapy						
1	Irinotecan HCl	Camptosar	DNA topoisomerase I inhibitor	Metastatic colorectal cancer	06/14/1996	(138)
2	Oxaliplatin	Eloxatin	Organoplatinum alkylating agent	Colorectal cancer (in combination with leucovorin and 5-FU)	08/09/2002	(139)
Passive immunotherapy (Ab based)						
3	Cetuximab	Erbitux	EGFR-directed mAb	Colorectal cancer	02/12/2004	(140, 141)
4	Bevacizumab	Avastin	VEGF-A-directed mAb	Colorectal cancer	02/26/2004	(142)
5	Panitumumab	Vectibix	EGFR-directed mAb	Colorectal cancer	09/27/2006	(143, 144)
6	Pembrolizumab	Keytruda	PD-1 targeted Ab	Colorectal cancer (for (dMMR and MSI-H types)	23/05/2017	(145, 146)
7	Nivolumab	Opdivo	PD-1 targeted Ab	Colorectal cancer (for (dMMR and MSI-H types)	01/08/2017	(147)
8	Yervoy	Ipilimumab	CTLA-4 targeted Ab	Colorectal cancer (for (dMMR and MSI-H types)	10/07/2018	(148–150)

### 5.1 Colorectal cancer passive immunotherapy

MSI-H tumors usually experience infiltration of immune cells, including TCD4^+^ (TH1) cells and TCD8^+^ (156); however, the immune cells are functionally unresponsive. These cells (present in the tumor stroma) usually express immune checkpoints such as PD-1 and CTLA-4, which bind to ligands on the surface of tumor cells (such as PD-L1 and CD28) and suppress the function of immune cells, especially T cells (157). Also, as it was said, by reducing the expression of β2MG, these tumors express a low level of stable MHC-1 on the tumor cell surface and escape from being recognized by TCD8^+^ cells (158). However, MSI-L colorectal cancers experience less infiltration of immune cells into the tumor stroma and have low levels of PD-1 expression (immune cells) and PD-L1 expression (tumor cells) (159, 160). Therefore, it seems that antibodies called immune checkpoint blockers (ICb) based on tumor type can be used to a greater extent in treating MSI-H tumors than MSI-L tumors (157).

In addition to the importance of the above classification, there is another molecular classification (Consensus Molecular Subtype (CMS)) for colorectal cancer, and knowing these characteristics is important for choosing the type of immunotherapy (161). From a molecular point of view, colorectal tumors are divided into four groups. This division simultaneously considers tumor and immune cells’ main gene expression changes in the different environments mentioned. CMS1 is a group of CRC where many mutations are observed (161). They are synonymous with MSI-H type, and mutations associated with BRAF genes are frequently observed in these tumors, including about 1/7 of colorectal tumors (161). CMS2, also called the canonical type, comprises approximately 1/3 of CRC and is associated with mutations that activate the Wnt and Myc signaling pathways (12). CMS3 tumors are also called metabolic type and include 1/7 of colorectal tumors, which often have KRAS-related mutations and disrupt the metabolic pathways of cancer cells (12, 162). The last type and CMS4, also called mesenchymal type, constitute 1/4 of colorectal tumor cases and are associated with activation of the growth factor-β (TGF-β) pathway, increased stromal activation, angiogenesis, and inflammatory infiltration (12).

The importance of these classifications is that the tumor microenvironment of each of these classes is different and therefore requires different treatments (163). In terms of immunity, CMS2 and CMS3 tumors are called cold tumors, which are at a very low level in terms of immune system responses, and in fact, the immune response against these tumors is not well established (161, 164). However, CMS1 and CMS4 tumors are called hot tumors that have higher immune responses than the previous groups. But the noteworthy point is that CMS1 and CMS4 show different responses to treatments due to their features for the immunotherapy index (165). As mentioned before, the CMS1 group, which is similar to MSI-H, was diffuse immune tumor-infiltrating lymphocytes (TILs) rich in CD8^+^ and CD68^+^ macrophages (162), whereas CMS4 tumors differed with a different pattern of immune infiltration, including monocyte-derived cells, regulatory t cells (Treg), MDSCs, and TH17 cells (166). The main factor in immunosuppression in the CMS4 type is the production of TGF-β and its related mechanisms (167). A therapeutic strategy of combining selective TGFβ inhibitors with immune checkpoint blockers can reactivate a strong immune response in these models of colon cancer.

In addition to antibodies that play a role in inhibiting immune checkpoints, other antibodies, including antibodies against cytokines such as TGF-β, can reduce the suppression of tumor-related immune responses (168). Also, antibodies against specific tumor antigens can sometimes be used to increase the recognition of tumor cells by the innate immune system (169). In this model, antibody attachment to tumor cells can lead to the tumor cells’ apoptosis by mechanisms such as antibody-dependent cellular cytotoxicity (ADCC), performed by macrophages and NK cells, and complement-mediated cytotoxicity (CDC).

### 5.2 Colorectal cancer active immunotherapy

Active immunotherapy, which usually occurs by cell transfer, uses T cells and DCs for treatment. The use of T cells occurs in 3 ways. In the first case, tumor-infiltrated lymphocytes (TILs) are collected from the tumor site, expanded, and injected into the patient as an autograft (170). This action leads to an increase in the number of tumor-specific T cells. In the second type of t cell therapy, T cell receptors (TCRs) are changed using genetic engineering methods, and their ability to identify antigens related to tumor cells increases (171). Another approach that has attracted much attention is using T cells with chimeric antigen receptors (CAR-T cells) (16, 172). In this technology, the Scfv part of an antibody that identifies tumor-specific or associated antigens (TSA or TAA) and its combination with different intracellular domains involved in T cell activation signal transmission is usually used (173).

In some cases, the extracellular part of CAR consists of different parts of a receptor whose ligand is abundantly produced in tumor cells (174). However, the applications of CAR-T have various limitations, and it is a dynamic field in cancer treatment, which is still being researched (175). CAR-T cell-based treatment is often used in clinical trial studies (**Table 4**).

**Table 4:** Examples of CAR-T cell clinical trials as novel therapeutics in colorectal cancer.

Study Name	Intervention Model	Estimated Enrollment	Antigen	Route	Phase	Dose	Date	NTC number	Major findings
NKG2D CAR-T Cells to Treat Patients With Previously Treated Liver Metastatic Colorectal Cancer	Sequential Assignment	9	NKG2DL	Hepatic artery transfusion	Early Phase 1	NA	2022	NCT05248048	Recruiting
Hepatic Transarterial Administrations of NKR-2 in Patients With Unresectable Liver Metastases From Colorectal Cancer (LINK)	Sequential Assignment	1	NKG2DL	Hepatic transarterial administrations	Phase 1	3 time administration: 3 × 10^8^–3 × 10^9^ cells/d(3ds)	2017	NCT03370198	No Results Posted
CAR-T Hepatic Artery Infusions or Pancreatic Venous Infusions for CEA-Expressing Liver Metastases or Pancreas Cancer (HITM-SURE)	Single Group Assignment	5	CEA	1. Hepatic artery infusions 2. Pancreatic venous infusions	Phase 1	1 × 10^10^ cells/d	2016	NCT02850536	1. ↑ Overall survival time. 2. CAR-T safely and effectively target CEA-expressing LM and achieve anti-tumor activity (176).
Anti-CEA CAR-T Cells to Treat Colorectal Liver Metastases	Single Group Assignment	18	CEA	Intravenous infusion	Phase 1	1- 6×10^6^/kg	2022	NCT05240950	Recruiting
EGFR-IL12-CART Cells for Patients With Metastatic Colorectal Cancer (EGFRCART)	Single Group Assignment	20	EGFR	NA	Phase 1	NA	2018	NCT03542799	No Results Posted
EGFR CART Cells for Patients With Metastatic Colorectal Cancer	Single Group Assignment	20	EGFR	NA	Phase 1 Phase 2	NA	2017	NCT03152435	No Results Posted
Binary Oncolytic Adenovirus in Combination With HER2-Specific Autologous CAR VST, Advanced HER2 Positive Solid Tumors (VISTA)	Single Group Assignment	45	HER2	Intra-tumor injection	Phase 1	1–100 × 10^6^Cells (1d)	2018	NCT03740256	Recruiting
Treatment of Relapsed and/or Chemotherapy Refractory Advanced Malignancies by CART133	Single Group Assignment	20	CD133	NA	Phase 1 Phase 2	0.5–2 × 10^6^ cells/kg (2ds)	2015	NCT02541370	1. The 3-month disease control rate was 65.2%. 2. Repeated cell infusions provide a longer disease stability period 3. CD133+ cells elimination occurred after CART-133 infusions (177).
P-MUC1C-ALLO1 Allogeneic CAR-T Cells in the Treatment of Subjects With Advanced or Metastatic Solid Tumors	Sequential Assignment	100	MUC1	NA	Phase 1	NA	2022	NCT05239143	Recruiting
Autologous CAR-T/TCR-T Cell Immunotherapy for Malignancies	Single Group Assignment	73	c-Met	NA	Phase 1 Phase 2	NA	2018	NCT03638206	No Results Posted
αPD1-MSLN-CAR T Cells for the Treatment of MSLN-Positive Advanced Solid Tumors	Single Group Assignment	10	EpCAM	Intravenous injection	Early Phase 1	1×10^5^-3×10^6^aPD1 MSLN-CAR+ T cells/kg (1d)	2020	NCT04503980	No Results Posted

In the case of CRC, there are limitations in the use of CAR-T cells, which include the low chemotactic ability of these cells to migrate to the tumor site, the acidic environment resulting from the metabolism of tumor cells, induced hypoxia, and lack of nutrients (173, 178). In addition to these cases, the severe immunosuppressive microenvironment in CRC is also one of the treatment obstacles. Among these substances that suppress transplanted CAR-T cells are anti-inflammatory cytokines, anti-inflammatory cells including Tregs, MDSCs, and tumor-associated macrophages, as well as metabolites derived from tumor cells (such as kynurenine produced from tryptophan by IDO) (178, 179).

In the study conducted by Jie Xu et al., they produced a human epidermal growth factor receptor 2 (HER2) based CAR-T cell. This receptor also is expressed on the surface of many healthy cells. However, its expression level is higher in tumor cells such as ovarian, stomach, colorectal, breast, and lung cancer (180). This study showed that HER2-specific CAR-T could increase anti-tumor responses in the CRC mouse model and has a high therapeutic capacity (181).

In addition to T cells, under the influence of TME, DCs acquire different functional and phenotypic characteristics, which leads to their non-functionality in anti-tumor responses and even suppression of immune responses (182, 183). According to the characteristics of tumor microenvironment-infiltrated DCs (TIDCs), such as their maturation level and their interaction with other cells in the tumor environment, including TILs, they can have positive or negative effects on CRC prognosis (184, 185). In addition to TIDCs, in CRC patients, the number and function of circulating DCs are generally reduced, and the immature or progenitor phenotype is also associated with an increase (186, 187). In general, it can be said that a large population of TIDCs (checked using the S100 marker) is associated with a good prognosis and less metastasis in CRC (188, 189). It has also been shown that the higher number of mature TIDCs in CRC is associated with better disease prognosis and TH and CTL responses (as seen in MSI-CRC) (190). It has been shown that the therapeutic use of DCs expressing PD-L1 leads to an increase in the lymph infiltration of TCD8^+^ cells to the CRC site and is associated with increased patient survival (191). Both circulating DCs and TIDCs produce cytokines and anti-inflammatory factors, including VEGF, IL-10, and TGF-β, which ultimately suppress T-cell responses (184). Also, in these cells, the expression level of some genes, including genes related to COX-2 and HMGB1, increases and helps to suppress the immune system’s responses (185). The important point is that tumor cells affect DCs by producing various factors such as CCL2, CXCL5, CXCL1, and VEGF. Therefore, if dendritic cells are isolated from CRC patients and autologously transplanted to patients after expansion and differentiation, they can exert strong anti-immune responses against the tumor by stimulating T cells (192). Another approach is to use the stimulating factors of DC responses and the processing and presenting of their antigens by *in situ* administration of their stimulating molecules, such as CpG, FLT3L, TLR, and STING agonists (124, 193).

## 6 Colorectal cancer combination therapy

Considering the TME and the complex behavior of tumor cells in the face of different treatments, it seems that monotherapy cannot achieve the desired results. As mentioned earlier, researchers had reached this conclusion many years ago and therefore used the combination of different chemotherapy drugs along with radiotherapy and surgery. With the emergence and increase of treatments based on immunology and biology to prevent the many side effects of chemotherapy, these treatments became more desirable options. In this way, researchers are interested in using multiple immunotherapies with fewer side effects to reduce the dose of chemotherapy drugs and help eradicate tumor cells.

### 6.1 Cytokine combination with chemotherapy

In the meantime, many achievements have been made that show that immunotherapy increases the effectiveness of chemotherapy. Studies have shown the existence of interactions between 5-FU and IFN-α in increasing cytotoxicity for different cancers (194). In a study by Laurent et al., 5-FU and IFN-α were used to treat colorectal cancer (194). The results of this study show that IFN-α, in combination with 5-FU, increases the amount of DNA single-stranded and double-stranded breaks in colorectal cancer cells *in vitro* by modulation of converting enzymes for anticancer prodrugs (194). Other studies also showed that IFN-γ could increase the activity of enzymes related to 5-FU anabolism (TP and UP) and thus help to remove more tumor cells by increasing the active form of this drug (195). Although the experimental results were promising, the combined use of 5-FU and IFN-α in clinical studies could not significantly affect patients’ survival rate or tumor removal (196). For this reason, the combined use of this drug stopped.

### 6.2 Combination of immune checkpoint blockades and chemotherapy

According to the treatment experiences of immune checkpoint blockades with chemotherapy drugs, these drugs seem to increase immune system cells’ capacity for anti-tumor responses (197). For example, it has been shown that using 5-FU can lead to apoptosis of MDSCs in the TME, which leads to the removal of immune inhibition induced by these cells (198, 199). In another way, the use of oxaliplatin, which leads to the apoptosis of tumor cells (199), causes the appearance of various antigens and their removal by DCs for the pancreas to T cells and leads to an increase in tumor-specific responses (200, 201). In one study, an *in vivo* assay using an immune checkpoint blockade mouse colon cancer model showed that an antitumor response was induced in the combined use of oxaliplatin with immune checkpoint inhibitors and resulted in increased survival in this model (157). In addition, 5-FU and atezolizumab (202), a humanized antibody against PD-L1, were used in a clinical trial (202). To use this combination, patients are first treated with the FOLFOX chemotherapy combination; then, they are treated with 5-FU and atezolizumab (202). However, the results of this study showed that adding this therapeutic combination does not affect the progression-free survival (PFS) and overall survival (OS) of patients.

### 6.3 Combination of monoclonal antibodies with chemotherapy

**Figure 1:**
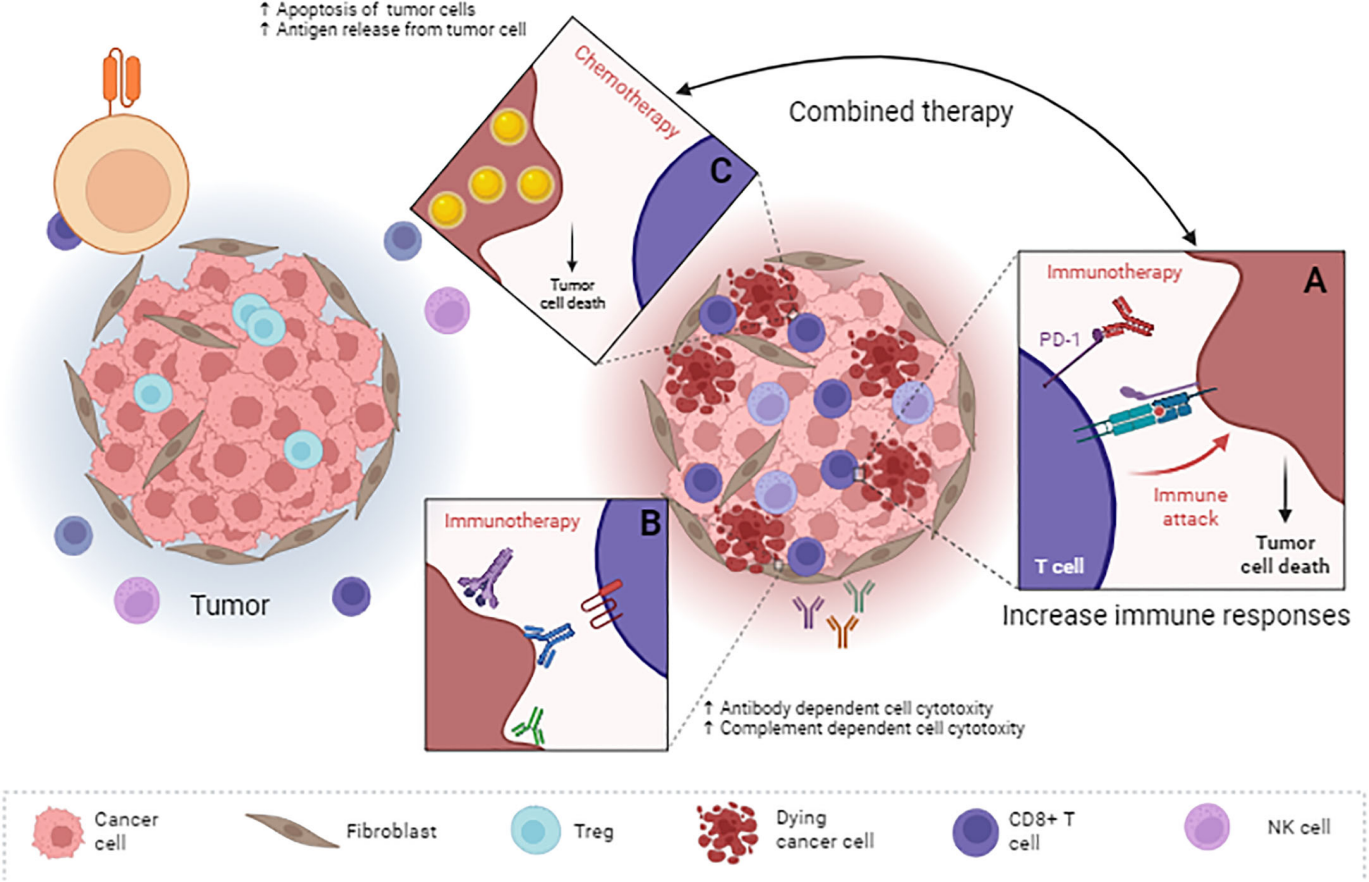
Combined use of chemotherapy and antibodies in the treatment of colorectal cancer. Antibodies can have different functions by targeting different tumor antigens. Some of them, such as immune checkpoint inhibitors **(A)** such as anti-PD-1 Ab and anti-CTLA-4 Ab, increase the response of immune cells, and some, by targeting a specific antigen **(B)**, leading to the activation of ADCC or CDC. Chemotherapy can lead to apoptosis of tumor cells and the release of various types of antigens derived from tumor cells **(C)**. On the one hand, the release of these antigens leads to an increase in the maturation of antigen-presenting cells. On the other hand, using immune checkpoint inhibitors leads to activating more immune system cells to deal with the tumor. In fact, these types of therapeutic compounds affect the tumor from different pathways and do not allow tumor recurrence after radiation or chemotherapy to the tumor cells. ADCC, Antibody-dependent cell cytotoxicity; CDC, Complement-dependent cell cytotoxicity.

Today, most patients with mCRC are treated with a first-line biologic agent, usually monoclonal antibodies against vascular endothelial growth factor (VEGF) or epidermal growth factor receptor (EGFR), depending on their RAS mutation status (203). As shown in **Table 5**, bevacizumab is a humanized anti-angiogenic antibody used to treat various tumors as a front line in combination with chemotherapy (208). The binding of this antibody to its ligand (VEGFR) leads to the suppression of new angiogenesis and normalization of blood vessels, which allows T cells to penetrate the tumor and activate them effectively (209, 210). According to those mentioned earlier, FOLFOXIRI and bevacizumab (targeting VEGF-A) in combination with atezolizumab (targeting PD-L1) were used in a clinical trial in the front line with chemotherapy, and the results obtained from it with the therapeutic use of bevacizumab and chemotherapy have been compared (211). The number of patients participating in this study was 218 patients, the results of which show that the PFS for patients receiving combined treatment (about 13 months) was higher compared to those receiving bevacizumab and chemotherapy alone (11.5 months) (p=0.07) (211). This change was not significant. Further studies have used FOLFOX combined with bevacizumab and nivolumab versus FOLFOX and bevacizumab for the treatment of CRC to investigate the effect of chemotherapy combined with immunotherapy in a clinical trial study (212). The primary endpoint of PFS was not met in this study. However, patients receiving FOLFOX combined with bevacizumab and nivolumab showed a higher PFS rate than patients receiving FOLFOX and bevacizumab after 12 months (212). Also, higher and more durable immune response rates were observed in the combined treatment compared to FOLFOX and bevacizumab, which indicates that the combined treatment of chemotherapy and tumor adenoma targeting with two antibodies that target different pathways has a higher therapeutic efficiency (**Figure 1**).

**Table 5:** Examples of Ab-based immunotherapy in combination with chemotherapy in clinical trials as novel therapeutics in colorectal cancer.

Study Name	Intervention Model	Estimated Enrollment	Drug	Phase	Date	NTC number	Major findings
LEAC-102 for Advanced Colorectal Cancer	Single Group Assignment	30	LEAC-102 500mg capsule and FOLFOX+Bevacizumab/Cetuximab	Phase 1 Phase 2	2016	NCT02826837	No Results Posted
Reolysin in Combination With FOLFOX6 and Bevacizumab or FOLFOX6 and Bevacizumab Alone in Metastatic Colorectal Cancer	Parallel Assignment	109	1. FOLFOX + Bevacizumab + Reolysin 2. FOLFOX + Bevacizumab	Phase 2	2012	NCT01622543	The addition of Reolysin to FOLFOX6/bevacizumab increases the patient’s overall response rate (204).
Efficacy of FOLFOX+Bevacizumab in Combination With Irinotecan in the Treatment of Metastatic Colorectal Cancer (CHARTA)	Parallel Assignment	250	1. Oxaliplatin, 5FU/LV, Bevacizumab 2. 5FU/LV, Oxaliplatin, Bevacizumab, Irinotecan	Phase 2	2011	NCT01321957	↑ Survival of patients (205).
Sequential Treatment Strategy for Metastatic Colorectal Cancer (ITACa)	Parallel Assignment	350	1. FOLFIRI or FOLFOX +Bevacizumab 2. FOLFIRI or FOLFOX 3. FOLFIRI or FOLFOX + CETUXIMAB 4. FOLFIRI or FOLFOX + BEVACIZUMAB and CETUXIMAB	Phase 3	2013	NCT01878422	Adding bevacizumab to standard first-line chemotherapy did not benefit progression-free survival, overall survival, and response rate (206).
2nd-line Treatment of Metastatic Colorectal Cancer (BEVATOMOX)	Parallel Assignment	83	1. Bevacizumab, oxaliplatin, and 5FU combination 2. Bevacizumab, oxaliplatin, and raltitrexed combination	Phase 2	2012	NCT01532804	Terminated No Results Posted
Neoadjuvant Treatment With mFOLFOXIRI Plus Cadonilimab (AK104) Versus mFOLFOX6 in Locally Advanced Colorectal Cancer (OPTICAL-2)	Parallel Assignment	82	1. mFOLFOXIRI + Cadonilimab 2. mFOLFOX6	Phase 2	2022	NCT05571644	Not yet recruiting
FOLFOXIRI + Bev + Atezo vs FOLFOXIRI + Bev as First-line Treatment of Unresectable Metastatic Colorectal Cancer Patients (AtezoTRIBE)	Parallel Assignment	201	Different combination of: Bevacizumab Irinotecan Oxaliplatin L-Leucovorin 5-fluorouracil Atezolizumab	Phase 2	2018	NCT03721653	↑ Progression-free survival in patients (207).
A Study of Biomarker-Driven Therapy in Metastatic Colorectal Cancer (mCRC) (MODUL)	Parallel Assignment	609	Different combination of: Cetuximab FOLFOX induction regimen Fluoropyrimidine (5-FU/LV or capecitabine) Atezolizumab Vemurafenib Bevacizumab Trastuzumab Pertuzumab Cobimetinib 5-FU/LV	Phase 2	2014	NCT02291289	N/A
Study of Pembrolizumab Treatment After CYAD-101 With FOLFOX Preconditioning in Metastatic Colorectal Cancer	Sequential Assignment	24	Different combination of: CYAD-101 FOLFOX Pembrolizumab	Phase 1	2021	NCT04991948	Recruiting
Chemotherapy and Immunotherapy as Treatment for MSS Metastatic Colorectal Cancer With High Immune Infiltrate (POCHI)	Single Group Assignment	55	Different combination of: Capecitabine Oxaliplatin Bevacizumab Pembrolizumab	Phase 2	2020	NCT04262687	Recruiting
Tucatinib Plus Trastuzumab and Oxaliplatin-based Chemotherapy or Pembrolizumab-containing Combinations for HER2+ Gastrointestinal Cancers	Sequential Assignment	120	Different combination of: Tucatinib Trastuzumab Oxaliplatin Leucovorin Fluorouracil Capecitabine Pembrolizumab	Phase 1 Phase 2	2020	NCT04430738	Recruiting

Among other studies that have dealt with the combination of chemotherapy and immunotherapy, we can refer to the study of GOIRC-03-2018 in phase II, in which the combination of triple chemotherapy (FOLFOXIRI) with bevacizumab and nivolumab in patients with CRC containing mutations Noted in RAS/BRAF (213). The results of this study show that the combination of FOLFOXIRI with bevacizumab and nivolumab is safe and has shown promising results; that’s why this group has continued its work in phase 3, the results of which have not been published yet. Also, in another clinical trial, although the POCHI trial (NCT04262687) is currently investigating the combination of CAPOX and bevacizumab with pembrolizumab as first-line treatment in eligible patients with MSS mCRC who have a high immune infiltrate, and the results are expected to show goodness in patients (214).

Cetuximab, a chimeric antibody (mouse V and human FC), is an IgG1 antibody that can increase the elimination of tumor cells by increasing ADCC and the expression of MHC-2 molecules on the surface of DCs (215, 216). It has been approved as part of the treatment regimen of CRC patients in combination therapy. In this way, to investigate the effectiveness of cetuximab in combination treatment with chemotherapy, treatment with cetuximab in combination with FOLFOX was used (phase 2 clinical trial), and it has been shown that this combination has potential therapeutic effects in selected patients (217, 218). The results of phase 3 clinical trial (TAILOR study) related to the therapeutic use of the combination of cetuximab and FOLFOX show that all the relevant clinical objectives and endpoints have been met cetuximab in combination with FOLFOX is an effective standard care first-line treatment regimen approved for patients with mCRC (219). Also, a study that investigates the therapeutic efficacy of adding avelumab to FOLFOX and cetuximab shows that this addition does not have a specific adverse effect (220). However, adding avelumab to the above combination did not reach its first endpoint, and no significant improvement was observed in the patient’s condition.

### 6.4 Combination of CAR-T cells with chemotherapy

**Figure 2:**
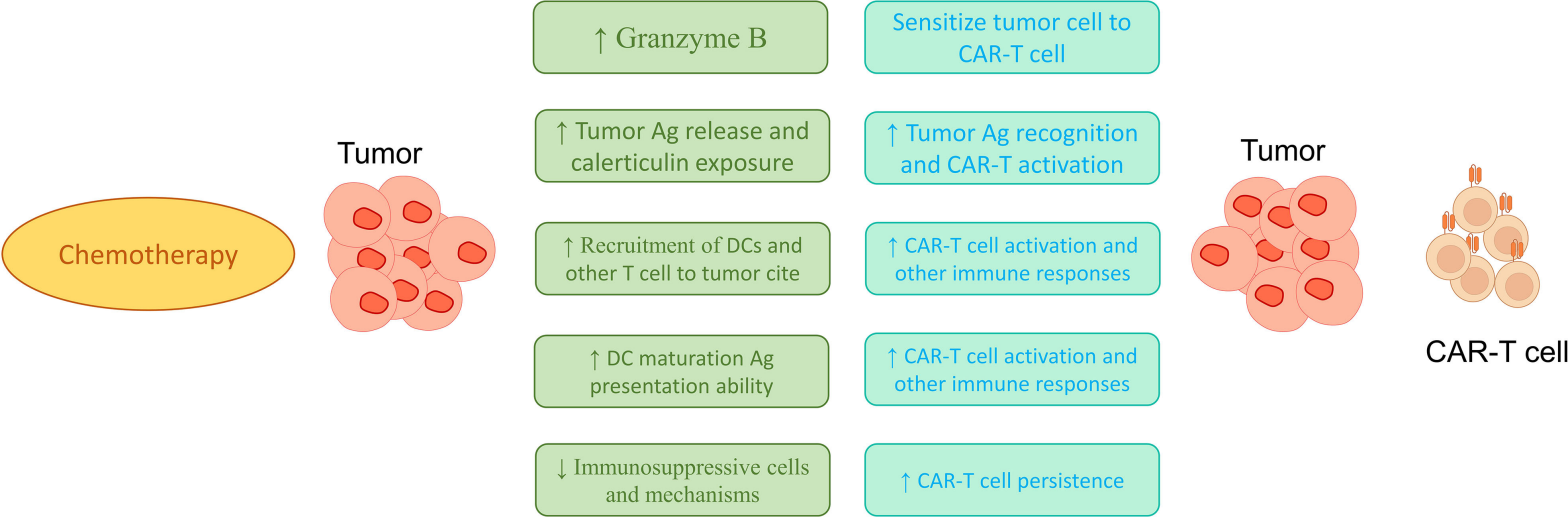
Possible mechanisms in increasing the effectiveness of treatments based on combinations of CAR-T cell and chemotherapy. As shown in the figure, the use of chemotherapy can increase the recruitment of immune cells, increase the maturation of dendritic cells, increase the activation of immune cells at the tumor site, and reduce the suppressive responses of immune cells to increase the potential therapeutic function of CAR-T cells.

It seems that by modulating the phenotype and abundance of blood leukocytes, chemotherapy could facilitate the production of the most effective CAR-T cell products. Also, by increasing the activity of circulating CD8+ T lymphocytes and rebuilding the effective memory population, chemotherapy can strengthen immune system responses (221). However, chemotherapy’s effect on other immune system cells, such as neutrophils, is harmful and can sometimes lead to neutropenia. Also, the results of various studies show inconclusive data about the effect of chemotherapy on TCD4^+^ cells (**Figure 2**) (222). Interestingly, Bellone et al. found induced and spontaneous IFN-γ release by TCD8^+^ cells after each course of combined chemotherapy with cisplatin, gemcitabine, and 5-fluorouracil (up to 4 cycles) are unchanged in comparison with pre-treatment values, indicating preservation of effect function throughout the treatment course (221, 223, 224). Therefore, it seems that the use of chemotherapy before transferring the CAR-T cells can provide suitable conditions in the body for the activity of these cells. However, during our search in the literature until March 2023, no study simultaneously used chemotherapy drugs and CAR-T cells to treat colorectal cancer. We recommend that researchers also use this method to treat CRC because chemotherapy can increase the therapeutic efficiency of CAR-T cells based treatments.

## 7 Conclusion and future perspective

As mentioned in many articles and based on the opinion of scientists active in the field of tumor treatment, therapies based on a drug usually cannot overcome the complex TME created by tumor cells. Regarding chemotherapy, due to its side effects, the lower the dose of the drug, the better the patient’s health and the fewer side effects. As discussed throughout this review, tumors use different methods and pathways to overcome various checkpoints that inhibit tumor growth. Also, many studies have shown that, in many cases, the use of a single treatment can lead to tumor resistance to that treatment. For example, cancer cells can become resistant to chemotherapy drugs by expressing some membrane pumps and creating new strains of tumor cells resistant to the given treatment. Also, using antibodies against immune checkpoints (For example, anti-PD-1 Ab) can lead to compensatory expression of other immune checkpoints (LAG-3 expression) on the surface of tumor cells (225). Therefore, it seems that combinational treatments that simultaneously target several different mechanisms related to the growth and immunosuppression induced by the tumor can be of great help in the treatment of tumors. As we know, the primary system that ultimately leads to tumor eradication is the immune system. Therefore methods based on activating this system more and more specifically against tumor cells can help eradicate the tumor faster. One of the ways to get the proper treatment and, at the same time, get the appropriate treatment is to use immunotherapy along with chemotherapy. As discussed in Section 6, in many studies, the use of this combination has helped improve patients’ health. But it is worth noting that in some cases, no significant change in the improvement of the condition of patients was observed in the combined treatment group and single treatment with chemotherapy. The critical point about these treatments is that the combination of chemotherapy and the CAR-T cells in colorectal cancer was not observed in the literature. While theoretically and in clinical applications, this combination is highly effective in improving patients’ conditions in other tumors. It seems that the use of new compounds, such as small molecules, chemotherapy, and immunotherapy, can affect this tumor treatment. However, more studies are needed to investigate the efficacy and safety of combination treatments.

## Author contributions

All authors contributed to the article and approved the submitted version.
